# Localization of Functions in the Cerebral Cortex

**Published:** 1883-07

**Authors:** 


### LOCALIZATION OF FUNCTIONS IN THE CEREBRAL CORTEX.

From the results of experiments on dogs, Bochfontaine concludes that Flourens was correct in ascribing vicarious functions to the cerebral convolutions. At one time electrical stimulation of a particular surface area *a* may, for example, be followed by secretion of the sub-maxillary gland or by some definite movement of a limb, while the same stimulus applied to other regions of the cerebral surface has no such consequences. In half an hour or forty-five minutes the region *a* will, however, cease to react to stimuli, while some other area *b*, previously inexcitable, becomes irritable, and its stimulation is followed by the same phenomena as previously the stimulation of *a*. The author suggests that the gray rind is itself not capable of electrical excitation, and that the result is always due to direct stimulation of subjacent medullated nerve-fibres. A bundle of such fibres, all with the same peripheral connection, may subdivide in the brain, and end in three or four different regions of its surface: to this assumption he adds the further gratuitous one, that only one cerebral division of the nerve-fibre bundle is excitable at any one moment.—*Arch. physiol. norm. Path.*

